# *AHA1* regulates cell migration and invasion via the EMT pathway in colorectal adenocarcinomas

**DOI:** 10.1038/s41598-021-99375-w

**Published:** 2021-10-07

**Authors:** Dasom Kim, Ji Wook Moon, Dong Hwa Min, Eun Sun Ko, Bokyung Ahn, Eun Sun Kim, Ji-Yun Lee

**Affiliations:** 1 Department of Pathology, Korea University College of Medicine, 73, Goryeodae-ro, Seongbuk-gu, Seoul, 02841 Republic of Korea; 2 Department of Biomedical Science, Korea University College of Medicine, Seoul, Republic of Korea; 3 BK21 FOUR Convergence & Translational Biomedicine Education Research Center, Department of Anatomy, Korea University College of Medicine, Seoul, Republic of Korea; 4 Department of Pathology, Asan Medical Center, University of Ulsan College of Medicine, Seoul, Republic of Korea; 5 Department of Internal Medicine, Korea University College of Medicine, Seoul, Republic of Korea

**Keywords:** Cell biology, Biomarkers, Oncology, Cancer, Gastrointestinal cancer, Metastasis, Oncogenes, Tumour biomarkers

## Abstract

The progression of colorectal cancer (CRC) has been well studied and understood with the development of molecular and genetic techniques. However, specific marker(s) that could be used to predict lymph node (LN) involvement, which is the most important prognostic factor for CRC, have not been identified so far. Our previous study, in which network analysis of LN(+) and LN(−) CRC gene expression was carried out with data obtained from the Cancer Genome Atlas, led to the identification of *AHA1*. *AHA1* is a co-chaperone activator of the Hsp90 ATPase activity. However, the role of *AHA1* expression in cancer cells is still unclear. To investigate how *AHA1* expression regulates the cancer cell progression and/or metastasis of human CRC, the expression levels of *AHA1* and Hsp90 were examined in 105 CRC tissue samples and compared with those in paired normal tissue. The RNA expression levels of *AHA1* and Hsp90aa1, but not Hsp90ab, were significantly higher in cancer tissues than in adjacent paired normal tissues (p = 0.032 and p = 0.0002, respectively). In particular, *AHA1*, but not Hsp90aa1 and Hsp90ab, was closely associated with the TNM stage, LN stage, and tumor metastasis (p = 0.035, p = 0.012, and p = 0.0003, respectively). Moreover, the expression of *AHA1* was not only higher in the CRC cell lines than in the normal colon fibroblast cell line but was also associated with the progression of these CRC cell lines. Overexpression of *AHA1* in SW480 cells increased, whereas suppression of *AHA1* expression in HCT116 cells reduced cell migration and invasion through the regulation of Snail, E-cadherin, pSRC, and pAKT, which are associated with EMT signaling. Taken together, our study suggests that *AHA1* contributes to the metastatic advantage of human CRC.

## Introduction

Colorectal cancer (CRC) is a high incidence and high prevalence disease worldwide, which influence on human health^1^. The development and progression of CRC from adenoma into cancer are systemic process, which has been well understood^2^. With advances in molecular and genetic technologies, our understanding of the molecular mechanisms through which genetic changes, such as genomic instability and alterations, in DNA lead to a normal mucosa turning into CRC, has expanded^3^. Molecular genetic studies in CRC, in respect of tumorigenesis, have discovered several meaningful genes and pathways that can be used in the early diagnosis of CRC. Nevertheless, the tumor node metastasis (TNM) stage is still major prognostic factor in CRC^4,5^. Stage II and III cancers are primarily differentiated based on nodal (N) stage, pointing out the importance of lymph node (LN) involvement in tumor prognosis. Currently, the N stage is determined by the pathological examination of LNs obtained during surgery. However, sometimes pathological examination, even though its usefulness, can make mistake by under-staging, resulting in lost opportunity for adjuvant chemotherapy, and a higher risk of tumor recurrence in patient^6,7^. This makes searching other factor or/and method for prediction or diagnosis of lymph node involvement extremely important for patient care.

In our previous study, we explored the possible diagnostic and/or prognostic marker(s) that might help investigate LN involvement in CRC, by comparing the networks of gene expression in LN(+) and LN(−) CRC using datasets from the Cancer Genome Atlas (TCGA) database (https://cancergenome.nih.gov/), and identified significantly different genes in the gene networks^8^. One of the genes, namely AHSA2, which belongs to the activator of the Hsp90 ATPase (AHA) family and is also known as a pseudogene, did not show any difference in expression between LN(−) and LN(+) in the subsequent investigation of the samples. However, another AHA family gene, namely *AHA1* (Ahsa1), which encodes protein that can activate the ATPase activity of Hsp90 as a co-chaperone^9,10^ was found to be upregulated in the LN(+) group compared to the LN(−) group. However, the role of *AHA1* in cancer, including CRC is very limited and still unclear. In this study, we investigated how *AHA1* affects cell migration, invasion, and the epithelial mesenchymal transition (EMT) signaling pathway in CRC cells, as well as whether *AHA1* expression correlates with clinical pathological characteristics such as TNM stages and MIS, to investigate its potential as a prognostic marker of CRC.

## Results

### *AHA1* is upregulated in progressive and metastatic CRC patients

**Figure 1:**
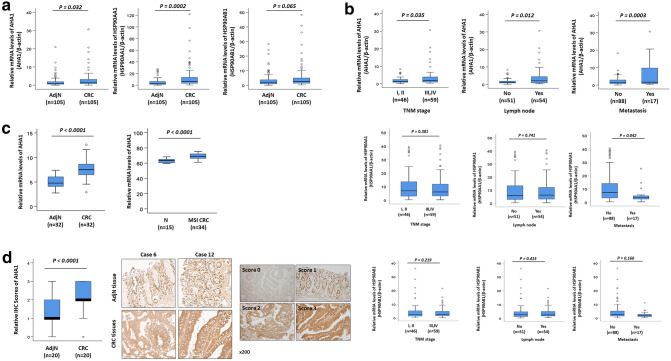
*AHA1* is up-regulated in progressive and metastatic CRC patients. (**a**) The mRNA expression of *AHA1* and HSP90AA1, but not of HSP90AB1, was significantly higher in CRC tissues compared to that in paired adjacent normal tissues. (**b**) The higher mRNA expression of *AHA1*, but not HSP90AA1 and HSP90AB1, was associated with the progressive clinical characteristics, such as the TNM stage, LN involvement, and metastasis, in CRC patients. (**c**) The mRNA expression of *AHA1* was significantly higher in 32 CRC tissues compared to that in 32 paired normal colonic mucosa tissues in GSE8671 datasets, as well as in 32 MSI CRC tissues, compared to that in normal colonic mucosa tissues in GSE24514 datasets. (**d**) *AHA1* expression was higher in CRC tissues, compared to that in adjacent normal tissues, which was scored by the scoring system after IHC stating and quantified by assessing staining intensity using Leica’s Aperio ImagScope program (V12.4.0.5043, IL, USA). Representative IHC-stained photomicrograph images with *AHA1* shown in CRC tissues, compared to adjacent normal tissues. *AdjN* adjacent normal specimen, *CRC* colorectal cancer specimen. Original magnification is ×200.

To confirm the mRNA expression levels of *AHA1*, Hsp90aa1, and Hsp90ab1 in CRC patients, qRT-PCR of these genes was performed for 105 paired CRC and adjacent normal tissues. The results showed that the expression of *AHA1* and Hsp90aa1 was significantly increased in CRC tissues compared to that in adjacent normal tissues (p = 0.032, p = 0.002, respectively. Figure 1a). Further statistical analysis revealed that the mRNA expression of *AHA1*, but neither Hsp90aa1 nor Hsp90ab1, is significantly correlated with the clinicopathological characteristics such as TNM stage (p = 0.035), lymph node metastasis (p = 0.012), and metastasis (p = 0.0003) (Fig. 1b, Table 1). In addition, mRNA expression of *AHA1* was analyzed using the GSE8671 and GSE24514 datasets^11,12^. The mRNA expression of *AHA1* was significantly increased in 32 CRC tissues compared to that in 32 paired normal colonic mucosa tissues from the GSE8671 datasets, as well as in 32 MSI CRC tissues, compared to that in 15 normal colonic mucosa tissues from the GSE24514 datasets (Fig. 1c). Results obtained from IHC staining of *AHA1* in CRC and adjacent normal tissues from 20 CRC patients were consistent with the mRNA expression data, in which *AHA1* expression was significantly increased in tumors (average score = 2.15) compared to that in adjacent normal tissues (average score = 1.35, p < 0.0001) (Fig. 1d). However, survival analysis using public dataset of colorectal adenocarcinoma did not showed significant association of *AHA1,* HSP90AA1, and HSP90AB1 RNA expression level with survival (Supplementary Fig. S1)^13^. These results suggest that *AHA1* may plays an important role in CRC progression and metastasis, however further investigation is necessary.

**Table 1:** Clinicopathological characteristics of CRC patients, and the mRNA expression levels of genes.

Characteristics	No. of cases	mRNA expression of Aha1 (%)		mRNA expression of HSP90AA1 (%)		mRNA expression of HSP90AB1 (%)	
		Median (range)	*p*-value	Median (range)	*p*-value	Median (range)	*p*-value
Adjacent normal	105	1.80 (± 0.27)	**0.032**	5.17 (± 0.61)	**< 0.001**	3.44 (± 0.45)	0.065
Colorectal cancer	105	2.89 (± 0.42)		12.50 (± 1.83)		5.09 (± 0.76)	
**Age (years)**			0.278		0.641		0.669
≤ 60	28	3.65 (± 1.11)		11.07 (± 2.66)		4.54 (± 1.35)	
> 60	77	2.61 (± 0.42)		13.02 (± 2.31)		5.28 (± 0.92)	
**Gender**			0.172		0.203		0.455
Female	39	3.64 (± 0.92)		15.54 (± 3.34)		5.83 (± 1.24)	
Male	66	2.44 (± 0.39)		10.70 (± 2.13)		4.64 (± 0.98)	
**Differentiation**			0.554		0.846		0.814
Well	29	2.20 (± 0.41)		14.07 (± 4.23)		5.86 (± 1.84)	
Moderate	73	3.19 (± 0.58)		11.79 (± 2.02)		4.76 (± 0.83)	
Poorly	3	1.99 (± 1.27)		14.47 (± 11.75)		5.47 (± 3.73)	
**Location**			0.739		0.621		0.983
Colon	72	2.79 (± 0.44)		11.88 (± 2.08)		5.10 (± 1.01)	
Rectum	33	3.09 (± 0.95)		13.84 (± 3.68)		5.06 (± 1.07)	
**Size (cm)**			0.072		0.48		0.242
≤ 6	77	2.42 (± 0.42)		15.62 (± 2.26)		7.20 (± 1.01)	
> 6	28	4.14 (± 1.08)		12.15 (± 2.96)		3.60 (± 0.66)	
**TNM stage**			**0.035**		0.34		0.487
I, II	46	1.88 (± 0.28)		14.49 (± 3.02)		5.69 (± 1.27)	
III, IV	59	3.67 (± 0.71)		10.95 (± 2.25)		4.61 (± 0.94)	
**Lymp node**			**0.012**		0.6		0.745
0	51	1.80 (± 0.26)		13.49 (± 2.76)		5.34 (± 1.15)	
1, 2	54	3.91 (± 0.77)		11.56 (± 2.44)		4.84 (± 1.02)	
**Invasion**			0.199		0.253		0.252
No	85	2.62 (± 0.39)		13.52 (± 2.21)		5.51 (± 0.93)	
Yes	20	4.01 (± 1.50)		8.17 (± 1.82)		3.27 (± 0.62)	
**Metastasis**			**< 0.001**		0.092		0.179
No	88	2.23 (± 0.26)		13.85 (± 2.14)		5.54 (± 0.90)	
Yes	17	6.28 (± 2.10)		5.49 (± 1.51)		2.74 (± 0.65)	

*TNM* tumor, lymph node, metastasis.

### High expression of *AHA1* correlates with progressiveness of colon cancer cells

**Figure 2:**
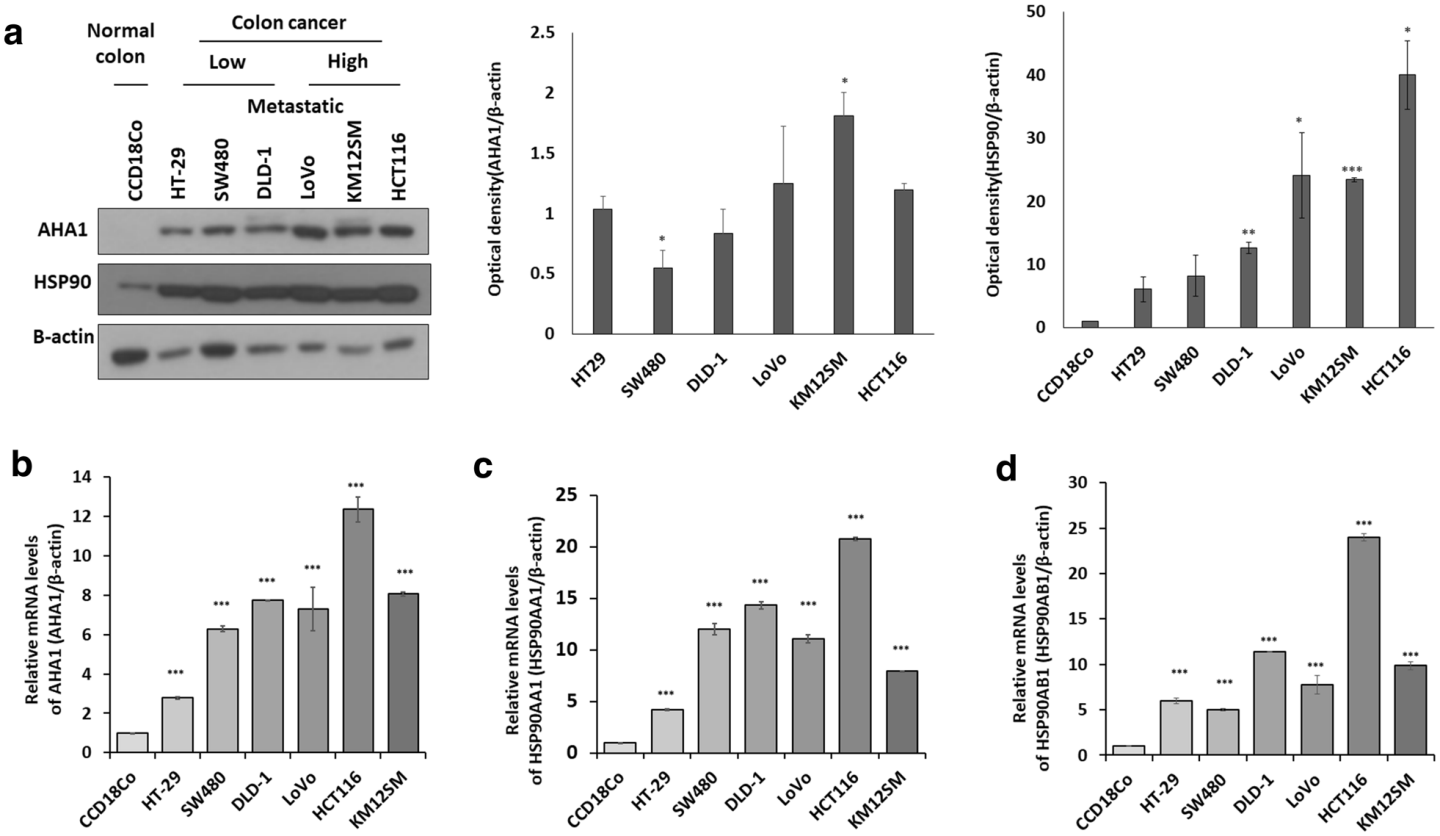
Expression of *AHA1* is associated with progressiveness of colon cancer cells. (**a**) The protein expression of *AHA1* and HSP90 was higher in the CRC cells than in normal colon fibroblast cells (CCD18Co), as well as in high metastatic CRC cells (KM125M, and HCC116) than in low metastatic CRC cells (SW480, and DLD-1), which was examined with western blotting. The densitometry quantification of the western blot determined using Image J software (Ver. 1/52n, NIH). (**b**–**d**) The mRNA expression of *AHA1*, HSP90AA1, and HSP90AB1, was higher in the CRC cells than in normal colon fibroblast cells (CCD18Co), which was detected with qRT-PCR. *p < 0.05, **p < 0.01, ***p < 0.001 compared with control.

The endogenous expression levels of *AHA1*, Hsp90aa1, and Hsp90ab1 were examined in normal colon fibroblast cells (CCD18Co), early-stage colon cancer cells (HT-29), middle grade colon cancer cells (SW480, DLD-1), and highly metastatic colon cancer cells (Lovo, KM12SA, and HCT-116), using western blot and qRT-PCR analyses. The results showed that not only did all colon cancer cells have increased *AHA1* expression compared to normal colon fibroblast cells (CCD18Co) but also the tendency of more progressive colon cancer cells to have a higher expression of *AHA1* (Fig. 2a,b). On the other hand, the expression of Hsp90aa1 and Hsp90ab1 increased in colon cancer cells, regardless of the cancer grade, compared to that in normal colon fibroblast cells (CCD18Co) (Fig. 2c,d).

### *AHA1* controls the migration and invasion of colon cancer cells

**Figure 3:**
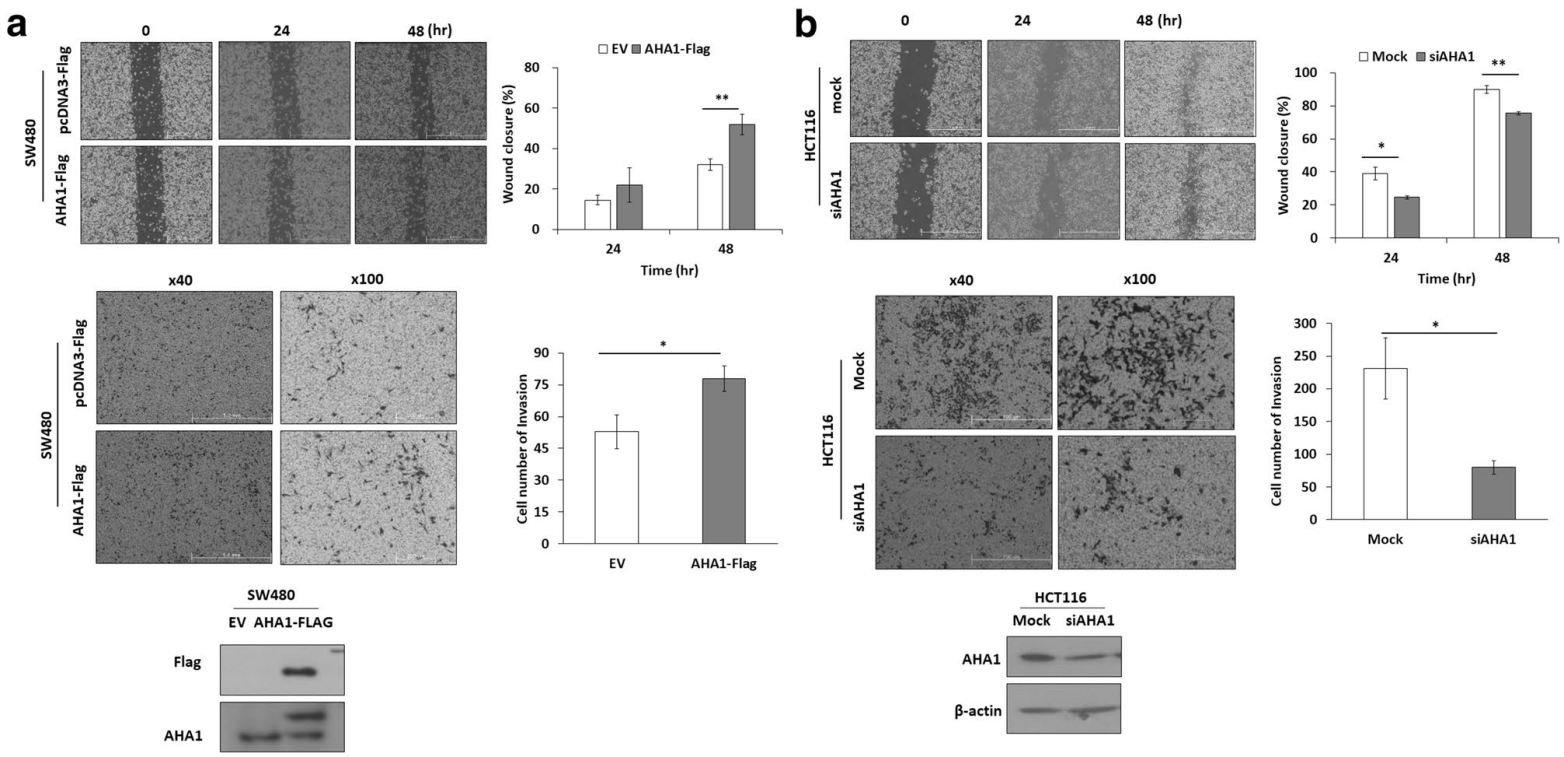
*AHA1* expression is associated with cell migration and invasion in CRC cells. (**a**) Overexpression of *AHA1* by transfection of *AHA1*-Flag for 24 h in SW480 enhanced cell migration and invasion. (**b**) Downregulation of *AHA1* by transfection of si*AHA1* for 48 h in HCT116 decreased cell migration and invasion. Cell migration was determined with wound healing assay for the indicated time point, and the degree of migration was quantified by calculating the area of migrated cells using the image processing software, ImageJ (Ver. 1/52n, NIH, Bethesda, MD, USA). Transwell assay was used to examine cell invasion. The representative images show membrane-associated cells stained using the Hemacolor rapid staining solution. *p < 0.05, **p < 0.01, ***p < 0.001 compared with control.

To investigate whether *AHA1* affects the proliferation, migration, and invasion of colon cancer cells, cell counting, wound healing, and transwell invasion assays were performed in SW480 and HCT116 cells with different expression levels of *AHA1*. To verify that *AHA1* enhances cell migration and invasion abilities, overexpression of *AHA1* by exogenously introduced *AHA1*-flag in SW480 cells, and knockdown of *AHA1* by siAHA1 in HCT116 cells, were performed. SW480 cells with overexpression of *AHA1* showed earlier wound closure as well as significantly increased cell migration across the membrane coated with Matrigel, and HCT116 cell with downregulation of *AHA1* showed opposite results, compared to that in the control after 24 h and 48 h (Fig. 3a,b). These results suggest that *AHA1* enhances the migration and invasion of colon cancer cells.

### *AHA1* regulates EMT signaling in colon cancer cells

**Figure 4:**
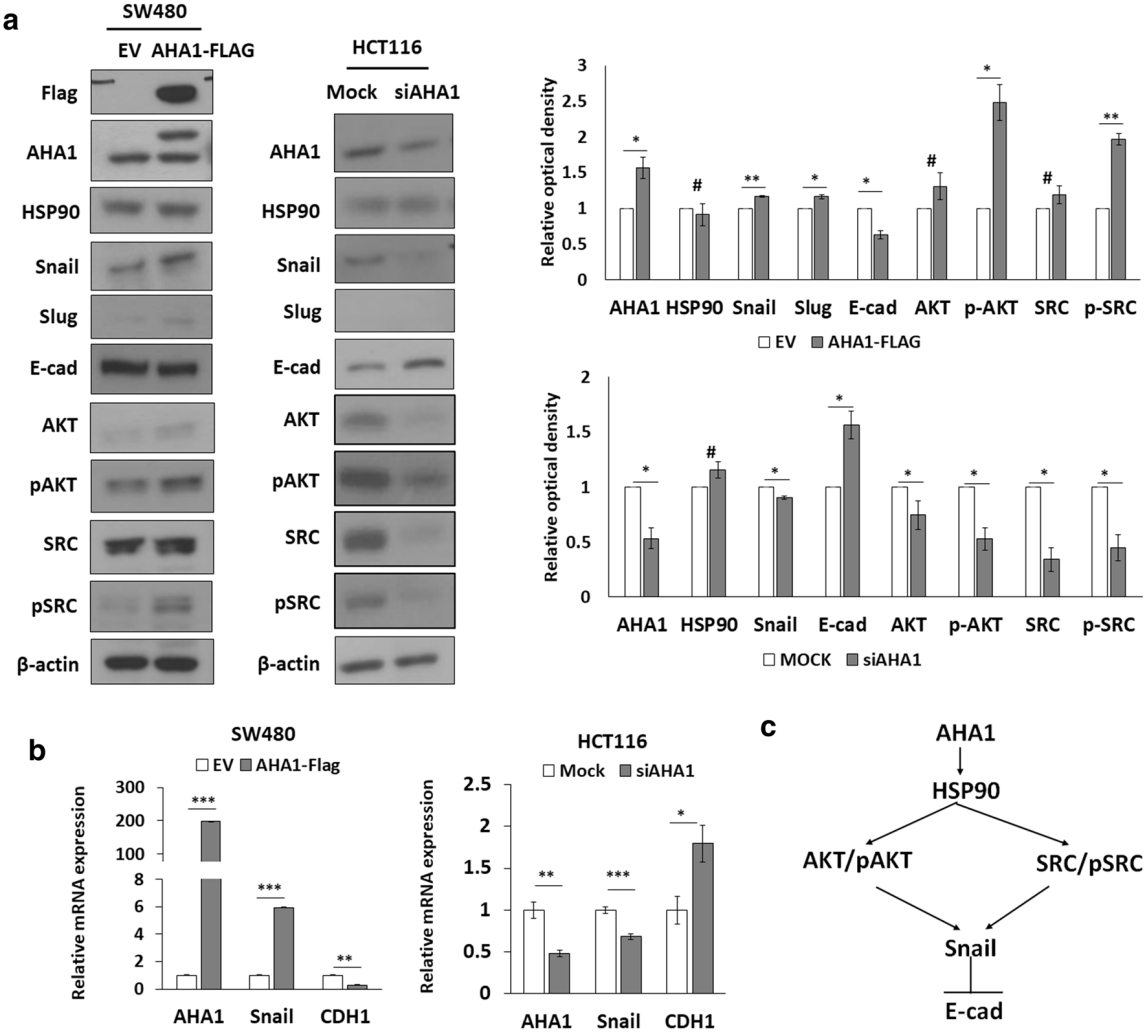
*AHA1* regulates EMT signaling through Snail, E-cadherin, pAKT, and pSRC. (**a**,**b**) Overexpression of *AHA1* by transfection of *AHA1*-Flag for 24 h in SW480 induced a decrease in the levels of E-cadherin, and an increase in the levels of Snail, pAkt, and pSrc expression. Downregulation of *AHA1* by transfection of si*AHA1* for 48 h in HCT116 induced an increase in the levels of E-cadherin, and a decrease in the levels of Snail, pAkt, and pSrc expression. Expression of proteins was determined with western blotting (**a**), and that of mRNA was determined using qRT-PCR (**b**). The densitometry quantification of the western blot determined using Image J software (Ver. 1/52n, NIH). *p < 0.05, **p < 0.01, ***p < 0.001, ^#^p ≥ 0.05 compared with control. (**c**) Schematic diagram of *AHA1* regulated signaling.

To identify the molecular pathways associated with *AHA1* in colon cancer cell migration and invasion, various molecules were examined with western blotting and qRT-PCR. Overexpression of *AHA1* by exogenously introduced *AHA1*-flag in SW480 decreased E-cadherin expression and increased Snail, pAkt, and pSrc expression. On the other hand, downregulation of *AHA1* by si*AHA1* in HCT116 increased E-cadherin expression and decreased Snail, pAkt, and pSrc expression (Fig. 4a,b). These results indicate that *AHA1* regulates cell migration and invasion via the EMT signaling pathway through Snail and E-cadherin, as well as phosphorylation of Akt and Src in colon cancer cells (Fig. 4c).

## Discussion

*AHA1* encodes a protein that activates the ATPase activity of Hsp90, leading to an increase in its chaperone activity^9,10^, and competes with inhibitory co-chaperones, such as Tsc1 and Fnip1, for binding to Hsp90aa1^14,15^. Numerous *AHA1*-interacting proteins have been identified, which are involved in a variety of intracellular bioprocesses, including DNA maintenance, chromatin structure, translation, nucleocytoplasmic transport, and vesicle transport, among others, pointing to the participation of *AHA1* in diverse Hsp90-regulated biological pathways to facilitate Hsp90 chaperone functions^16,17^. Most of the previous studies have focused on interactions of *AHA1* with other chaperones of the Hsp90, and the consequent effects of the Hsp90 chaperone complex on various biological functions. Only limited research has so far been carried out on the association of *AHA1* expression with, and its role in various diseases, especially cancer^18–22^. In this study, we focused on how *AHA1* expression is associated with CRC cancer cell migration and invasion, which is one of the hallmarks of cancer cell progression. Our study showed that *AHA1* was significantly overexpressed in tumor tissues, compared to that in adjacent normal tissues, in CRC patients, as well as in the analysis of GEO datasets. In addition, overexpression of *AHA1*, but not Hsp90, was significantly associated with higher TNM stage, LN involvement, and metastasis, implying its association with the progressiveness of CRC. To verify this finding, in vitro cell migration and invasion analysis was performed in context of *AHA1* expression, using colon cancer cells. A comparison of *AHA1* expression in colon cancer cells showed that more aggressive cells, such as HCT116 and KM125M, expressed more *AHA1*, along with Hsp90, than less aggressive cells, such as SW480, HT29, and normal colon fibroblast cells (CCD18Co). Also, HT-29 and SW480 cells were MSS, whereas DLD-1 was MSI^23,24^. Since *AHA1* (RNA) expression is higher in DLD-1 cells than SW480, it may be interesting to further investigate association of *AHA1* and MSI/MSS status. The relationship between *AHA1* expression and migration and invasion was evaluated by overexpression and knockdown of *AHA1*, which showed that overexpression of *AHA1* in SW480 increased, and knock-down of *AHA1* in HCT116 decreased cell migration and invasion, a finding consistent with previous reports on osteosarcoma^20–22^. To determine which molecular signaling pathways are associated with *AHA1* in colon cancer cell migration and invasion, various molecules were examined with western blotting after overexpression or knockdown of *AHA1* in the colon cancer cells. Snails and E-cadherin, which are well-known EMT markers, as well as pSrc, and pAkt, showed changes in their levels in response to alteration of *AHA1* expression. These results showed that EMT signaling, which is the most well-known mechanism of cancer metastasis in context of colon cancer cell migration and invasion, is regulated by *AHA1*. Src is a proto-oncogene non-receptor tyrosine kinase protein, whose activation is known to promote the survival, angiogenesis, proliferation, and invasion pathways in cancer, including CRC^25^. Elevated c-Src levels have also been reported to be correlated to advanced stages of the tumor, tumor size, metastatic potential of tumors in CRC, and control of the PI3K/AKT signaling pathway^26,27^, which indicates that both Src and Akt are directly or indirectly associated with EMT signaling^28–30^. Both Akt and c-Src are also known to be a client protein of Hsp90^31,32^. Studies have revealed that Hsp90 also binds to and stabilizes Snail, which is a driver of EMT signaling during tumor progression through E-cadherin, in response to DNA damage^33^. It is known that Hsp90 and its client proteins play important roles in cancer development through the regulation of various signaling proteins, including EMT signaling, and not only Hsp90, but also co-chaperons considered as an attractive target for anticancer therapy^34,35^. In addition, it has been reported that the increased expression of *AHA1* alters the activity of Hsp90 client proteins and the phosphorylation status of key signaling proteins, including Akt, resulting in enhanced kinase activity^36–38^, and affects the efficacy of Hsp90 inhibitors, such as Tanespimycin (17-*N*-allylamino-17-demethoxygeldanamycin, 17-AAG), in cancer cells. This points to a potential therapeutic strategy to increase the sensitivity of cancer cells to Hsp90 inhibitors, by disrupting the Hsp90-*AHA1* complex and targeting *AHA1*^38^.

Taken together, our results show that the expression of *AHA1* regulates CRC cell migration and invasion via EMT signaling, through pAkt, pSrc, Snail, and E-cadherin, which possibly occurs via regulation of Hsp90 activity in the HSP90/*AHA1* complex. Activation of Hsp90 ATPase activity is the only known biochemical function of *AHA1*; although we have not confirmed the ATPase activity in this study, we cannot rule out alternative or additional effects of *AHA1*. In addition, *AHA1* may serve as a potential prognostic marker associated with LN involvement and metastasis, as shown in our results, even though further verification is needed with a greater number of patient samples.

## Methods

### Tumor samples

One hundred and five cases of CRC and paired adjacent normal tissues were obtained from the Department of Colorectal Surgery, Korea University Medical Center. The diagnosis of CRC tissues was made based on pathology reports and histological evaluations. The tissues for this study were collected after obtaining approval from the Institutional Review Board of Korea University College of Medicine (IRB No. KU-IRB-13-84-A-1) and informed consent was obtained from all subjects or, if subjects are under 18, from a parent or legal guardian. Fresh tissue samples were frozen in liquid nitrogen after resection, and stored in a deep refrigerator at − 80 °C until use. Among these cases, patients who received preoperative treatment and those with no available paraffin block were excluded, and a total of 20 primary CRC and 20 metastatic CRC cases in patients, with or without metastasis, were reviewed. All hematoxylin-and-eosin (H&E)-stained slides of the cases were reviewed by pathologists and used for immunohistochemistry (IHC) staining. Clinicopathologic data, including age, sex, tumor location, tumor size, grade, pathologic T (T) category, pathologic N (N) stage, lymphovascular invasion, perineural invasion, LN status, distant metastasis, and resection marginal status, were reviewed. The TNM stages were adjusted to the specifications of the 8th American Joint Committee on Cancer Staging Manual. All experimental protocols were approved by the Institutional Review Boards of Korea University Anam Hospital (K2018-2161-001). All methods were carried out in accordance with the relevant guidelines and regulations. The clinicopathologic features of CRC patients are summarized in Table 1.

### Analysis of Gene Expression Omnibus (GEO) datasets and clinical information

We used two datasets (GSE8671 and GSE24514), downloaded from the National Center of Biotechnology Information Gene Expression Omnibus (GEO) database (https://www.ncbi.nlm.nih.gov/gds/), to compare the expression of target genes between CRC tissues and normal tissues. GSE8671 included the transcriptome data of 32 colorectal adenomas with paired adjacent normal mucosa^11^. GSE24514 included the expression profiles of 34 MSI colorectal cancers and 15 normal colonic mucosae^12^. We performed survival analysis in colorectal cancer for *AHA1*, HSP90AA1, and HSP90AB1 using OncoLnc (http://www.oncolnc.org/). OncoLnc is a web-based analysis tool that can analyze survival correlation using The Cancer Genome Atlas (TCGA) data^13^.

### Cell cultures and reagents

One normal colon fibroblast cell line (CCD18Co) and six colon cancer cell lines, HT-29, SW480, DLD-1, LoVo, HCT116, and KM12SM, were obtained from the American Type Culture Collection (Manassas, VA, USA). CCD18Co cells were cultured in Eagle’s minimum essential medium, and eight CRC cells were cultured in RPMI 1640 or Dulbecco’s modified Eagle’s medium, supplemented with 10% fetal bovine serum (FBS, WELGENE) and 1% penicillin/streptomycin (WELGENE) at 37 °C in a humidified atmosphere containing 5% CO_2_. The pcDNA3-Flag-*AHA1* plasmid was constructed using the one-step SLIC method^39^. The oligo ribonucleotide sequences of human *AHA1* siRNA (si*AHA1*) were as follows: 5′-AUU GGU CCA CGG AUA AGC U-3′ (sense) and 5′-GUG AGU AAG CUU GAU GGA G-3′ (antisense) (Shanghai GenePharma Co. Ltd., China).

### Quantitative real time polymerase chain reaction (qRT-PCR)

Total RNA was isolated from cells using TRIZOL (Invitrogen, Carlsbad, CA, USA). cDNA was synthesized, using a reverse transcription kit (Labopass, Cosmo Genetech, Seoul, South Korea) according to the manufacturer’s instructions, from the total RNA extracted from frozen tissue and cell lines. qRT-PCR was conducted using gene-specific primers with SYBR Green Q Master (Labopass) on an ABI 7500 Real Time PCR System (Applied Biosystems, Warrington, UK). The following PCR primers were used: *HSP90AA1* sense: 5′-ACC CAG ACC CAA GAC CAA CCG-3′, antisense: 5′-ATT TGA AAT GAG CTC TCT CAG-3′; *HSP90AB1* sense: 5′-GTG CAC CAT GGA GAG GAG-3′, antisense: 5′-ATT AGA GAT CAA CTC CCG AAG-3′; *AHA1* sense: 5′-CAG AGG GAC ACT TTG CCA CCA-3′, antisense: 5′-CTC GAC CTT CCA TGC ACA GCT-3′; Snail sense: 5′-GAC CCC AAT CGG AAG CCT AAC TA-3′, antisense: 5′-AGC CTT TCC CAC TGT CCT CAT CT-3′; CDH1 sense: 5′-CGG GAA TGC AGT TGA GGA TC-3′, antisense: 5′-AGG ATG GTG TAA GCG ATG GC-3′; β-actin sense: 5′-AGA GCT ACG AGC TGC CTG AC-3′, antisense: 5′-AGC ACT GTG TTG GCG TAC AG-3; glyceraldehyde 3-phosphate dehydrogenase (*GAPDH)* sense: 5′-ACC CAC TCC TCC ACC TTT GA-3′, antisense: 5′-CTG TTG CTG TAG CCA AAT TCG T-3. The Ct values of the target genes were normalized to those of an endogenous reference genes (β-actin and *GAPDH*). Each gene was analyzed in triplicate in three independent experiments.

### Immunohistochemistry (IHC) staining for *AHA1*

IHC studies were performed on formalin-fixed, paraffin-embedded slides of 20 CRC and adjacent normal tissues, to determine the expression of *AHA1* in accordance with the manufacturer’s protocol, using rabbit polyclonal antibodies against *AHA1* and HRP-labeled goat anti-rabbit polyclonal secondary antibody (Abcam plc, Cambridge, MA). Counterstaining was carried out using hematoxylin. IHC results were scored, with an average cytoplasmic staining intensity of 0 (no expression), 1 (mild intensity), 2 (moderate intensity), and 3 (strong intensity), by a pathologist, and staining intensity was quantified using Leica Aperio ImagScope (V12.4.0.5043, IL, USA).

### Cell migration assay

Cell migration was studied using a wound-healing assay. Cells were seeded into 6-well plates and cultured to a confluent monolayer. The cell monolayer was scratched with a 200 μl sterile micropipette tip, and the wells were washed twice with phosphate-buffered saline (PBS) to remove detached cells. The cells were then cultured for indicated hours in RMPI-1640 supplemented with 2% FBS, to minimize cell proliferation during the period of assay^40^. The image of each scratch at the same location was captured after the indicated incubation time. The healed area was measured from the captured images using ImageJ (Ver. 1/52n, NIH, Bethesda, MD, USA).

### Transwell invasion assays

The invasiveness of cells was evaluated with an invasion assay using a Transwell device (CT-3422, 8 μm pore size, 6.5 mm diameter, Corning Life Science, USA) coated with Matrigel (BD, 356230, 100 μg/ml, 15 µl/well). Cells (1 × 10^5^) were seeded in the upper chamber of the Transwell device in serum-free media. The lower chamber of the transwell device was filled with cell growth medium containing 10% FBS as a chemoattractant. The invaded cells were fixed and stained with the Hemacolor rapid staining solutions (Merck, USA) for 5 min after removing the non-invading cells. The number of invaded cells was counted in five representative fields of the membrane under a light microscope (DP71, Olympus, Japan).

### Western blotting analysis

The proteins from cell lysates were separated using SDS-PAGE and transferred to Immobilon-P PVDF Membrane (IPVH00010, Millipore, USA). These membranes were cut based on the size of the target protein (Supplementary Fig. S1) including loading control, and subsequently probed with the indicated primary antibodies and incubated with the appropriate goat anti-rabbit IgG or goat anti-mouse IgG (Cell Signaling Technology) secondary antibody conjugated with horseradish peroxidase (HRP), before signal detection using the enhanced chemiluminescence (ECL) system (Translab, Daejeon, South Korea) according to the manufacturer’s instructions. The blots were cut prior to hybridisation with antibodies (Supplementary Fig. S2). The primary antibody against *AHA1* (GTX102312) was purchased from GeneTex (CA, USA). The antibody against Flag (F1804) was purchased from Merck. The antibody against HSP90 (ab13492) was purchased from Abcam. The antibodies against E-cadherin (14472) and Slug (9585) were purchased from Cell Signaling Technology (Danvers, MA, USA). The antibodies against Snail (sc-271977) and β-actin (sc-47778) were purchased from Santa Cruz Biotechnology. The densitometry quantification of the western blot determined using Image J software (Ver. 1/52n, NIH). The result of gels images was cropped and the unprocessed original blots are included in the Supplementary Figs. S2, S3, S4.

### Statistical analysis

Results are indicated as mean ± standard deviation (SD) from at least three independent experiments. The significance of different mRNA expression values among CRC and adjacent normal tissues was determined by one-way analysis of variance (ANOVA) and the paired t-test using IBM SPSS Statistics version 25.0 (IBM Inc., Chicago, IL, USA). Comparisons between groups were made using a two-tailed Student’s t-test or ANOVA test. If the p-value obtained with ANOVA was < 0.05, the p-values between the groups were compared with post-test, Bonferroni, and Tukey HSD. p-values ≤ 0.05 were considered statistically significant.

## Supplementary Information

Supplementary Figures.
