# Supplementary material for: AHA1 regulates cell migration and invasion via the EMT pathway in colorectal adenocarcinomas
Source: Sci Rep. 2021 Oct 7;11:19946. doi: 10.1038/s41598-021-99375-w (PMC8497578; doi:10.1038/s41598-021-99375-w)
Supplement: Supplementary file 1 — Supplementary Figures. [file 41598_2021_99375_MOESM1_ESM.rtf]

AHA1 regulates cell migration and invasion via the EMT pathway in colorectal adenocarcinomas
Dasom Kim^*1,2^, Ji Wook Moon^*3^, Dong Hwa Min^1,2^, Eun Sun Ko^1,2^, Bokyung Ahn^4^, Eun Sun Kim^5^, Ji-Yun Lee^1^


Fig. S1.  Kaplan-Meier survival curve of AHA1(A), HSP90AA1(B) and HSP90AB1(C) analyzed using OncoLnc website (http://www.oncolnc.org/). Survival analysis was performed on 440 colon adenocarcinoma tissues and 158 rectal adenocarcinoma tissues obtained from TCGA by classifying them into high and low expression based on the median values of AHA1, HSP90AA1 and HSP90AB1. 

  

Fig. S2. The blots were cut prior to hybridization with antibodies. Example of membrane cut and blot of target protein to probe with the appropriate primary antibody. 


A


B


C


Fig. S3. Full-length blots of Fig.2a (A), and Fig3 (B), and Fig.4 (C) with loading control â-actin on the same blot. Red box indicated image used in figures. 
A


B


Fig. S4. Replicate blot images (red box) performed for the quantification of Fig 4. A. SW480 B. HCT116
